# Antioxidant and Antibacterial Activity of Four Tannins Isolated from Different Sources and Their Effect on the Shelf-Life Extension of Vacuum-Packed Minced Meat

**DOI:** 10.3390/foods12020354

**Published:** 2023-01-11

**Authors:** Chau Ngoc Minh Nguyen, Nilesh Prakash Nirmal, Yasmina Sultanbawa, Zyta M. Ziora

**Affiliations:** 1 Centre for Nutrition and Food Sciences, Queensland Alliance for Agriculture and Food Innovation, Health and Food Science Precinct, 39 Kessels Road, Coopers Plains, Brisbane, QLD 4108, Australia; 2 Institute of Nutrition, Mahidol University, 999 Phutthamonthon 4 Road, Salaya, Nakhon Pathom 73170, Thailand; 3 Institute for Molecular Bioscience, The University of Queensland, St Lucia, Brisbane, QLD 4072, Australia

**Keywords:** tannin, antioxidant, antibacterial, natural preservative, beef mince, quality control

## Abstract

Four tannin samples extracted from chestnut wood (tannin oenologique, TO), grape (tannin VR grape, TVG), oak gall (tannin galalcool, TG), and oak tree (tannin VR supra elegance, TE) were evaluated for antioxidant and antibacterial activity. The highest total phenolic content (TPC) values were observed in the order of TVG > TG > TE > TO (*p* < 0.05). The antioxidant activities of all samples were determined in terms of DPPH radical scavenging activity, reducing power, metal-chelating activity, and linoleic acid peroxidation assay. The antioxidant activities of all samples vary and no correlation was observed with the respective TPC values of each sample. Antibacterial activities indicate that all samples showed more or less inhibitory effects against selected Gram-positive and Gram-negative bacteria. Based on antioxidant and antibacterial activity, TO and TVG were selected for the beef mince quality preservation study during refrigerated storage. Both TO and TVG at two different concentrations, 0.25 and 0.5%, could cease the chemical and microbial changes as compared to the control sample. Although total viable count (TVC) did not show a significant difference, the H2S-producing bacteria count was lower in all samples treated with TO and TVG compared to sodium metabisulfite (SMS) and the control sample (*p* < 0.05). Therefore, TO and TVG could be promising natural food preservatives during refrigerated storage.

## 1. Introduction

Natural or plant-based antioxidants and antibacterials were recently in the loop of the food industry for the improvement of the shelf life and quality control of different food systems. In contribution to limiting food spoilage, many attempts have been made to extend the shelf-life of food. A few chemical compounds such as butylated hydroxy anisole (BHA), butylated hydroxytoluene (BHT), benzoic acid, and sodium metabisulfite (SMS) had been used to control intrinsic factors. Unfortunately, many detrimental side effects have been exposed to human health. For instance, BHA could potentially disrupt endocrine glands, resulting in body weight gain, delayed sexual maturation, slower sperm motility [1], and female breast cancer [2]. There is a rising need for greener and healthier preservatives in food. Natural additives such as plant extracts are preferred by consumers as they are perceived as clean, green technologies. It is not just a general perception, in scientific terms; natural additives are described as generally recognised as safe (GRAS). For instance, the addition of 0.3% cinnamon powder in apple juice can inhibit *Listeria monocytogenes* [3], *Salmonella typhimurium*, *Yersinia enterocolitica*, and *Staphylococcus aureus* [4]. Anise extract was proven to be highly effective on *Aspergillus flavus* [5,6]. The antioxidant and antibacterial properties of plants belong to the active phytochemical contents, which can be categorised into three classes: phenylpropanoids, isoprenoids, and alkaloids [7,8].

Phenylpropanoids are a group of diverse, important secondary products including flavonoids, stilbenes, and proanthocyanidins (also known as condensed tannins). They essentially are antioxidant, antibacterial, antiviral, and on top of that, anticancer [9]. Proanthocyanidins (condensed tannins) and hydrolysable tannins are characterised by the polymerisation degree of flavonoids bound together at position C4–C8 and C4–C6 [10], several hydroxyl groups and aliphatic moieties, and branch extensions [11]. Tannins are widely distributed in all plants as they play an important role in defence systems such as pesticide and growth regulation [12,13]. They are commonly found in leaves, bark, twigs, roots, seeds, and fruit [12]. Recently, there have been a few investigations looking into the bacteria-killing properties of tannins. The combination of low temperature (4 °C) and 200 mg/L of polyphenols in Merlot wine can clear out 90% of *L. monocytogenes* in 1 day and 90% of *E. coli* in 2.4 days using the fish meat model [14]. In another trial with lean cow meat, the same polyphenol extraction from Merlot wine could also reduce 90% of the population of *L. monocytogenes* in 2.5 days and *E. coli* in 1.5 days [14]. Noticeably, tannins are included in the polyphenol mixture extracted from Merlot in both trials, but not alone. 

Tannin oenologique (TO) is isolated from chestnut wood. A 10% portion of the chestnut mass is tannins [15]. The characterisation of chestnut tannins gives a composition of numerous tannin compounds: gallic acid, syringic acid, sinapaldehyde, syringaldehyde, hexahydrodiphenic acid, pentagallolglucose, ellagic acid, castalin, castalagin, and valoneic acid [16]. Tannin VR grape (TVG) is extracted from grape vines and is mostly found abundantly in wine. Grapes have a completely different tannin composition than chestnut wood tannins and only include the polymerisation of four main monomers: catechin, epicatechin, epicatechin-3-O-gallate, and epigallocatechin [17,18]. Tannin galalcool (TG) is derived from oak galls, which appear to be tumours on oak trees. The extraction from oak gall contained a significant amount of gallic acid and 47.2% of gall dried weight is tannins [19,20,21]. Tannin VR supra elegance (TE) is pressed out from the oak tree and composed of only two structures, proanthocyanidin and ellagin. Although only two compounds are claimed to be in the product, more compounds such as pentagalloyglucose, vescalagin/castalagin, vescagin/castalin, vescavaloneic/castavaloneic acid, and free gallic acid could be found in the crude extract of oak wood [22].

Meat and meat products, particularly minced meat, are highly perishable with limited shelf-life due to microbial and chemical deterioration. The normal shelf-life of minced meat in refrigerated storage is 5–7 days, while vacuum-packed minced meat can be stored for 21 days [23]. Further, it is reported that 40% of meat packaging has been done by vacuum sealing [24]. Therefore, the objectives of this study were to evaluate the antioxidant and antibacterial properties of tannin from four different sources including chestnut wood called tannin oenologique (TO); grape, named Tannin VR grape (TVG); oak gall, called tannin galalcool (TG); and oak tree, as Tannin VR supra elegance (TE). Additionally, the shelf-life extension effect of TO and TVG treatment on vacuum-packed beef mince during 21 days of refrigerated storage was evaluated. The physicochemical and microbial changes in the beef mince sample treated with TO, TVG, SMS, and control were determined during refrigerated storage.

## 2. Materials and Methods

### 2.1. Samples

Tannin oenologique (TO), tannin VR grape (TVG), tannin galalcool (TG), and tannin VR supra elegance (TE) powder samples (200 g) were provided by Laffort company. The chemical composition or major active component in each sample has not been studied in this experiment. All samples were stored at room temperature before use for experiments.

### 2.2. Determination of Total Phenolic Content

The total phenolic content (TPC) of samples was determined using the Folin–Ciocalteu reagent assay method. The stock solutions (5 mg/mL) of each sample were prepared in distilled water. Further, 50-time diluted samples were used for TPC analyses. Briefly, 25 μL of diluted extracts were mixed with 125 μL of Folin–Ciocalteu reagent (1:10 dilution). Then, 125 μL of sodium carbonate solution (7.5%) was added to the mixture and the reaction mixtures were incubated at 24 °C for 30 min [25]. The absorbance of reaction mixtures was recorded at 750 nm against a blank. Total phenolic content was calculated from standard gallic acid solutions (0–100 mg/L) used under the same conditions, and concentrations were expressed as g of gallic acid equivalents (GAE) per 100 g of dried powder. 

### 2.3. Determination of Antioxidant Activities

#### 2.3.1. 2,2-Diphenyl-1-Picryl Hydrazyl (DPPH) Radical Scavenging Activity

DPPH solution (0.15 mM) was prepared by dissolving DPPH (Sigma-Aldrich, St. Louis, MO, USA) in 95% ethanol and stirring for 4 h at room temperature in the dark. Different concentrations of samples were used for analyses, including 5, 10, and 50 µg/mL. The reaction mixture was prepared by incubating samples and DPPH reagent (1:1) [25]. The mixture stood in the dark for 30 min at room temperature and was measured at 517 nm absorbance using a spectrophotometer (Genesys-6, Thermo-Scientific, Madison, Wisconsin, MA, USA). The sample blank at each concentration was prepared in the same manner except that ethanol was used instead of DPPH solution. Control was prepared with DPPH solution and ethanol without any sample. The percentage of the inhibition of DPPH radical was calculated from the following equation:

% Inhibition = (1 − Asample/Acontrol) × 100

where Asample is the absorbance of the sample reaction mixture and Acontrol is the absorbance of the control.

#### 2.3.2. The Ferric-Reducing Power Assay

Various concentrations of samples were prepared with phosphate buffer (0.2 M, pH 6.6). One millilitre of the sample at different concentrations (5, 10, and 50 µg/mL) was mixed with 1 mL of potassium ferricyanide (1%) and incubated at 50 °C for 20 min [26]. After incubation, 1 mL of trichloroacetic acid (10%) was added to the mixture, followed by centrifugation (Centrifuge 5804R, Eppendorf AG, Hamburg, Germany) at 2000× *g* for 10 min. The upper layer of the solution (1 mL) was mixed with distilled water (1 mL) and 0.1% ferric chloride (0.2 mL) and the absorbance was read at 700 nm using a UV-visible spectrophotometer. Higher absorbance of the reaction mixture indicated greater reducing power. 

#### 2.3.3. Metal-Chelating Assay

The metal-chelating activity of the sample was determined according to the method of Gursoy et al. [27]. Briefly, 2 mL of various concentrations (1, 2.5, and 5 µg/mL) of the sample in ethanol (95%) was added to a solution of 2 mM FeCl_2_ (0.05 mL). The reaction was initiated by the addition of 5 mM ferrozine (0.2 mL). Then, the mixture was shaken vigorously and left at room temperature for 10 min. The absorbance of the reaction was read at 562 nm. A blank sample consisting of a 2 mL sample solution with 2 mM FeCl_2_ (0.05 mL) and water (0.2 mL) without ferrozine was prepared. The control contained ethanol, FeCl_2_, and ferrozine. The inhibition percentage of ferrozine–Fe^2+^ complex formation was calculated by using the formula given below:

Metal chelating activity (%) = [(Acontrol − Asample)/Acontrol] × 100

where Acontrol and Asample are the absorbances of the control and sample, respectively.

#### 2.3.4. Linoleic Acid Peroxidation Method

The linoleic acid emulsion was prepared by mixing 0.28 g linoleic acid with 0.28 g Tween-40 in a 50 mL phosphate buffer (0.2 M, pH 7.0) [28]. Samples in an amount of 0.5 mL (1 mg/mL) in ethanol were mixed with linoleic emulsion (2.5 mL) and phosphate buffer (2.5 mL). The mixture was then incubated at 37 °C for 120 hrs. The control was prepared in the same manner except for the test sample. The peroxidation of the emulsion was determined using the thiocyanate method. Aliquots (0.1 mL) of the linoleic acid emulsion were drawn every 24 hrs to mix with 5.0 mL ethanol (75%), 0.1 mL ammonium thiocyanate (30%), and 0.1 mL FeCl2 (20 mM in 3.5% HCl). The mixture stood at room temperature for 3 min before the absorbance was read at 500 nm. The degree of peroxidation was calculated at 96 hrs by the following formula:

Degree of peroxidation = [1 − (Increase in absorbance of sample/Increase in the absorbance of control)] × 100

### 2.4. Antibacterial Analysis

Four strains of bacteria, including 2 Gram-positive strains (*Staphylococcus aureus*—ATCC 33591, *Listeria monocytogenes*—ATCC 19111), and 2 Gram-negative strains (*Escherichia coli*—ATCC 11775, *Pseudomonas aeruginosa*—ATCC 9626) were used for antimicrobial screening using 96-well plates. These species were chosen due to their reputation for food poisoning and also accessibility at the time. Different concentrations of extracts ranging from 0.005 to 5 mg/mL were used for analysis. The 6 middle rows were for 6 replicates, the first row only had the medium to confirm the sterilisation of the medium as well as the plate, and the last row only had the culture. All cultures were grown in a nutrient broth medium. After 24 h of incubating at 37 °C, starter cultures were adjusted to OD 0.5 at 540 nm against nutrient broth [29]. Amounts of 50 µL of culture, 100 µL of nutrient broth, and 50 µL of samples were pipetted into each well. Bacterial growth was read at 620 nm and the percentage inhibition of growth was calculated by the following formula:

% Inhibition = (AC − A)/AC × 100

AC is the optical density of the control

A is the optical density of the sample

Moreover, the minimum bactericidal concentration (MBC) was determined by streaking the MIC from the above plate to the nutrient agar plate. The growth of bacteria was observed after 24 h of incubation at 37 °C.

### 2.5. Effect of TO and TVG on the Quality of Beef Mince during Refrigerated Storage

From the antioxidant and antibacterial results of tannins, TO and TVG with high antioxidant activity and antibacterial activities were selected for the treatment of minced beef meat. According to the antibacterial activity result, maximum inhibition was observed at 1.25 and 2.50 mg/mL concentrations of tannin samples. Hence, these two concentrations were further selected for the treatment. However, to be effective in a real food system, a 2-fold concentration of TO and TVG solution was used, which was 2.5 and 5.0 mg/mL or 0.25 and 0.5 g per 100 g of minced meat. Moreover, there was no immediate colour effect on the meat sample from TO or TVG treatments.

#### 2.5.1. Preparation of Beef Mince Treated with TO and TVG

Minced beef (10 kg) was bought from a local supplier. The beef mince was mixed with TO and TVG powder (0.25 and 0.5% weight/weight ratio) by blending for 10 min. Another portion of beef mince was mixed with sodium metabisulphite (0.05%) as a positive control. Beef mince without any treatment was used as a control. Each batch was divided into 8 equal portions and placed separately in sterile, vacuumed plastic bags (200 g per bag). All samples were stored at a refrigerated temperature (4 °C) for 21 days. Samples (2 bags) of each treatment including control, TO, TVG, and SMS were taken every 7 days up to 21 days for chemical and microbiological analyses.

#### 2.5.2. Chemical Analyses

##### pH Measurement

pH measurement was conducted in the homogeneous mixtures of meat with distilled water in a proportion of 1:10, weight to volume [25]. 

##### Analysis of Thiobarbituric Acid Reactive Substances (TBARS)

One gram of meat was homogenised with 5 mL of TBA solution, which contained 0.0375% thiobarbituric acid, 15% trichloroacetic acid, and 0.25 M HCl [25]. Then, the mixture was incubated in a boiling water bath for 10 min. After cooling under running tap water, the mixture was centrifuged (4000× *g*) at 4 °C, for 20 min. The supernatant was measured against the blank at 532 nm. TBARS value was calculated from the standard curve of malonaldehyde (0–2 ppm) and results are expressed as mg of malonaldehyde per kg of sample.

##### Determination of Metmyoglobin (Metmb)

The contents of Metmb in ground beef were determined as described by Badr [30]. Briefly, 5 g samples were homogenised in 25 mL ice-cold 40 mM phosphate buffer (pH 6.8). Then, the homogenate was centrifuged at 4500× *g* for 30 min at 4 °C. The supernatant was filtered through Whatman filter paper and the absorbance was measured at 572, 565, 545, and 525 nm using a spectrophotometer. Then Metmb percentages were calculated based on corrected absorbance values, as follows:

% Metmb = [−2.51 × (A572/A525) + 0.777 × (A565/A525) + 0.8 × (A545/A525) + 1.098] × 100

#### 2.5.3. Colour Measurement

Colour parameters were directly evaluated on a transparent plastic bag surface during the storage time using a CIE colourimeter (Hunter associates laboratory Inc., Reston, VA, USA). The colour of the samples was read as *L (brightness) *a (redness) *b (yellowness) values [31]. The total difference in colour (∆E*) was calculated according to the following equation:

$$∆E*=\sqrt{{\left(L_{2}-{\;L}_{1}\right)}^{2}+{\left(a_{2}-a_{1}\right)}^{2}+{\left({\;b}_{2}-{\;b}_{1}\right)}^{2}}$$

where L_1_, a_1_, and b_1_ denote values at 0 days of storage time, and L_2_, a_2_, and b_2_ denote values at 21 days of storage time.

#### 2.5.4. Microbial Analyses

Ten grams of meat was transferred aseptically into a stomacher bag, which was, later, mixed with 90 mL of peptone (0.1%) and homogenised for 60 s at room temperature using a Lab Blender 400 from Seward Medical UK.

##### Total Viable Count

One millilitre of the suspension was used for serial dilutions (1:10 diluent) in 0.1% peptone water. Then, all dilutions were mixed with 10 mL of molten agar using the pour plate method. Plate Count Agar (PCA, CM0463, Oxoid, Basingstoke, UK) was used to determine the total viable count after incubation for 72 h at 30 °C [25].

##### H2S-Producing Bacteria Count

Iron agar (IA, CM 867, Oxoid, Basingstoke, UK) was used to determine bacteria that produce H2S. After settling 1mL of dilution, 10 mL of molten agar was added. Iron agar plates were incubated for 72 h at 30 °C [32]. Counting was conducted based on black colonies on red agar. Two replicates of appropriate dilutions were enumerated. Microbiological data were transformed into logarithms of 10 of the number of colony-forming units (CFU/g). 

### 2.6. Statistical Analysis

All experiments were performed in duplicate and analyses were conducted in triplicate unless mentioned elsewhere. A one-way analysis of variance (ANOVA) was performed to determine significant differences between treatments using Tukey’s post hoc (HSD) and Duncan’s range tests. All results are expressed as the mean ± standard deviation. Differences were considered significant at *p* < 0.05. The statistical analysis was conducted using an XLSTAT package (Microsoft Excel).

## 3. Results and Discussion

### 3.1. Antioxidant Activities of Tannin Samples

#### 3.1.1. Total Phenolic Contents

The total phenolic content in all samples varied with the different origin sources. The highest amount of phenolic compound was recovered from TVG, which was 71.25 g GAE/100 g powder, and the lowest TPC was observed in TO, with 35.82 g GAE/100 g powder (Table 1). TO from chestnut wood may contain only 10% polyphenols [15]. The second highest TPC was observed in TG with 61.14 g GAE/100 g powder followed by TE with only 51.75 g GAE/100 g powder. It was concluded that there were more polyphenols in grapes than in oak, oak galls, and chestnut wood.

#### 3.1.2. DPPH Radical Scavenging Activity

The IC50 value of each extract to scavenge the 50% DPPH radical is shown in Table 1. The lowest IC50 value indicates the highest DPPH radical scavenging activity. TO, with 4.03 µg/mL, shows the highest DPPH radical scavenging activity, followed by TG (6.23 µg/mL), TVG (15.26 µg/mL), and TE (20.25 µg/mL). The data for free DPPH radical scavenging do not correlate with total phenolic content. The TPC and DPPH scavenging activity of six different fruit residues, kinnow peel and seeds, litchi pericarp and seeds, grape seeds, and banana peel, showed no correlation with each other [33]. The fluctuating trend in TPC and DPPH radical scavenging activity was also noted in the same genus. Ten different types of soybeans were screened for phenolic content and antioxidant activity and it was concluded that there was no parallel correspondence between the two parameters [34]. Since the scavenging activity vastly depends on the capacity of giving away hydrogen, the answer may land in the ratio of hydrolysable tannins and condensed tannin contents in the extraction. Hydrolysable tannins are highly vulnerable to hydrolysis by acid or enzymes while condensed tannins or non-hydrolysable tannins are much more resistant to hydrolysis [35]. Therefore, hydrolysable tannins are more likely to give electrons. Hence, this result indicated that TO and TG could have more free hydroxyl groups compared to other extracts.

#### 3.1.3. Ferric-Reducing Power

**Figure 1:**
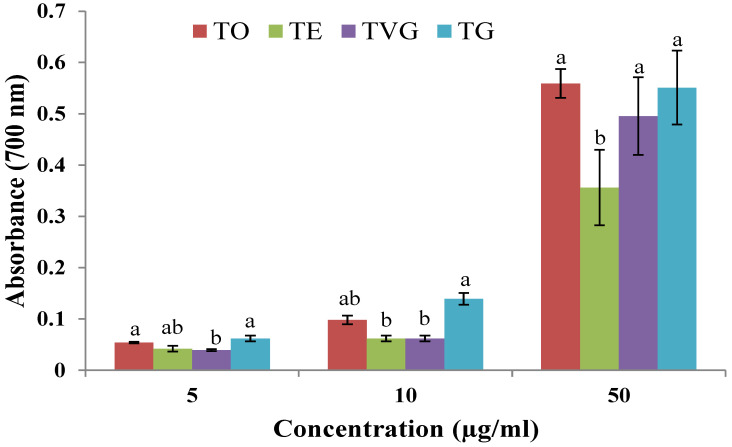
Ferric-reducing power of four different tannin isolates. TO: tannin oenologique; TVG: tannin VR grape; TG: tannin galalcool; TE: tannin VR supra elegance. Mean ± SD (N = 3). Means with different letters are significantly different (*p* < 0.05).

The reduction capacity may serve as an indicator of the antioxidant potential of a compound. In this test, the increase in absorbance indicates more reduced power. Increasing the concentration of extracts increases the absorbance of the reaction (Figure 1). The increase in absorbance was higher for TO and TG as compared to TE and TVG (*p* < 0.05). However, there was no statistical difference observed between TO, TG, and TVG at higher concentrations (*p* > 0.05). Results from ferric-reducing power correlate well with the DPPH radical scavenging activity of tannins. They share the same mechanism, which uses antioxidants to reduce the oxidants. Therefore, they may also share the same reason for antioxidant activities. It was hypothesised that the reducing power was dependent on linkage position and 3-O-gallolation degree [36]. For example, an oligomer constructed of epicatechins connecting at C4 to C8 was easier to oxidise than an oligomer built of catechins linking at position C4 and C6 [37]. However, three adjacent hydroxyl groups on the B-ring would not give myricetin higher antioxidant power; it would reduce it instead. This could point out that more hydroxyl group availability would not increase the power of giving electrons. This is the exact reason why higher total hydroxyl groups do not necessarily mean higher antioxidant activity [37].

#### 3.1.4. Metal-Chelating Activity

**Figure 2:**
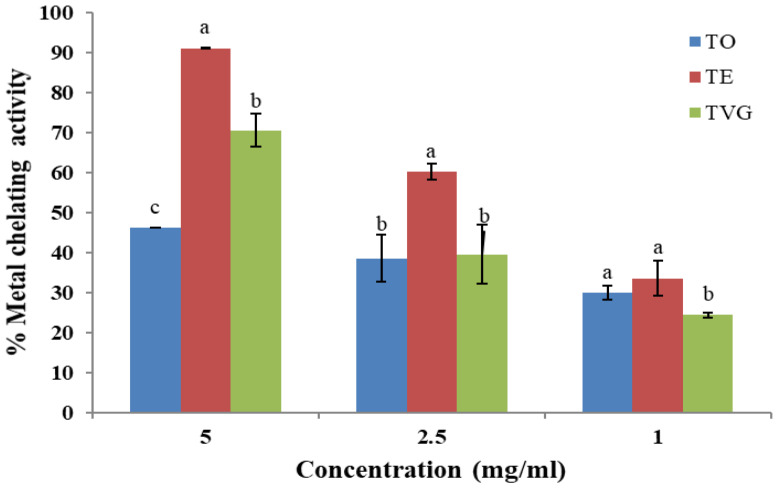
Metal-chelating activity of four different tannin isolates. TO: tannin oenologique; TVG: tannin VR grape; TE: tannin VR supra elegance. Mean ± SD (N = 3). Means with different letters are significantly different (*p* < 0.05).

The metal-chelating activity of TO, TE, and TVG at different concentrations is presented in Figure 2. Surprisingly, TE had the highest metal-chelating activity at all concentrations as compared to TO and TVG (*p* < 0.05). TG was not included in the figure because it needed 75 mg of TG to chelate 90% of the metal, which was not economical in terms of large-scale application. The second best at a concentration of 5 mg/mL was TVG, with 71% chelating activity. Although, at low concentrations (1 and 2.5 mg/mL), TO and TVG showed a similar percentage of chelating activity (*p* > 0.05). The solution of dried Vitis Vinifera grape in water showed much more effective metal-chelating activity, which was 95% at the concentration of 100 µg/mL [38]. This result indicates that dried fruit extract contains more active compounds than pure tannin extract. In contrast to reducing power, the glycosylation of flavonoid monomers will make metal-binding activity impossible [39]. This is actually in agreement with the conclusion in reducing power in which TG has the highest polymerisation degree compared to the others.

#### 3.1.5. Linoleic Acid Peroxidation

**Figure 3:**
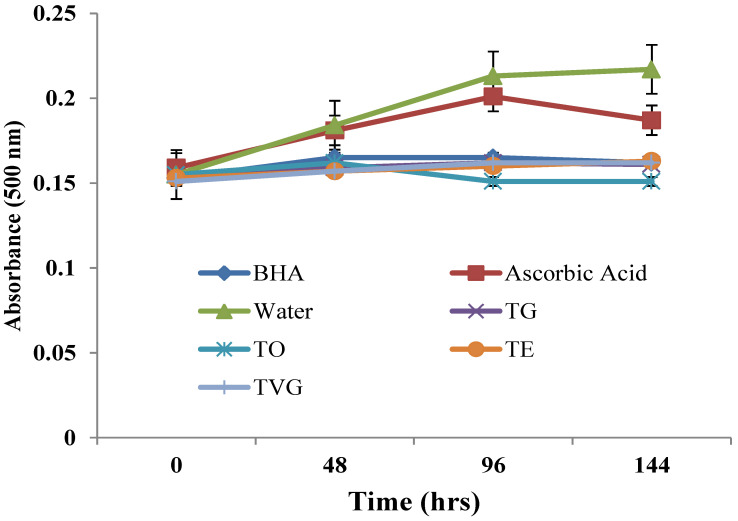
Linoleic acid peroxidation inhibitory activity of four different tannin isolates. TO: tannin oenologique; TVG: tannin VR grape; TG: tannin galalcool; TE: tannin VR supra elegance. Mean ± SD (N = 3). Means with different letters are significantly different (*p* < 0.05).

The linoleic acid peroxide inhibitory activity of tannin samples (1 mg/mL) during 144 hrs of incubation is presented in Figure 3. During 4 days of incubation, more colour was produced as the fatty acid started to break down, as shown by the control (water). Thus, a high absorbance value indicates a lower stabilising potential. In general, the four tannin extracts gave similar effects compared to each other (*p* > 0.05). All samples showed similar inhibitory activity as compared to BHA (100 ppm) on the last day. TO could only give a considerable effect as compared to other tannin samples. Moreover, all of them outperformed vitamin C in this matter. All curves were expected to drop at some point after 4 days due to the nature of the testing chemicals. When linoleic acid is not available anymore, the formation of peroxides cannot be continued. Then, there are no more oxidised products to react with ferrous chloride to form ferric chloride, which couples with thiocyanate to produce colour. Instead, all fatty acids were converted into stable end products that have no effect on ferrous chloride [40]. The percentage of inhibition of the linoleic acid peroxidation value of TVG was 83%, which was consistent with the value reported by Jayaprakasha et al. [40]. Overall, giving absolute protection, TO (1 mg/mL) would be a promising candidate to replace harsh chemical preservatives for oil and fat. 

### 3.2. Antibacterial Activity of Tannin Samples

The antibacterial activity of tannin samples was tested against two Gram-positive and two Gram-negative bacteria, as shown in Table 2. Four types of tannins at concentrations of 1.25 and 2.5 mg/mL could successfully stop *Listeria* from growing (100% inhibition) (*p* > 0.05). However, they did not exhibit the same impact on *S. aureus*, which is also Gram-positive. TVG and TE were able to eliminate the whole *S. aureus* population at a concentration of 2.5 mg/mL. TVG was still able to produce the same antibacterial activity at a lower concentration, 1.25 mg/mL, whereas TG and TO (2.5 mg/mL) inhibited 42% and 56% of *S. aureus* growth, respectively (*p* < 0.05). On the other hand, it was completely opposite in treatment with the Gram-negative bacteria. TG and TO were able to kill 100% of *E. coli* with the two highest concentrations, but TE and TVG hardly inhibited 50% of the growth of *E. Coli* (*p* < 0.05). Additionally, TO, TE, and TVG inhibited a large portion of the *p*. aeruginosa culture, more than 80% (*p* > 0.05), while TG showed only 44% (*p* < 0.05) inhibition at a 2.5 mg/mL concentration. Being the cause of severe disease with a high mortality rate, *L. monocytogenes* is always in the loop for food scientists and pharmacists. Moreover, the bactericidal test was able to determine that the lethal concentration for *Listeria* was 2.5 mg/mL for the four kinds of tannins. TVG, especially, was able to wipe out 100% *Listeria* at the lower concentration of 1.25 mg/mL (*p* < 0.05). TG and TO were not able to inhibit *S. aureus* at all at 1.25 mg/mL (*p* > 0.05), and the streak plate method confirmed that TG and TO had no bactericidal effect on *S. aureus*. However, the data correlated well between the inhibition and bactericidal activity of TE and TVG against *S. aureus*.

The results on *E. coli* and *p. aeruginosa* were quite intriguing. As mentioned in the metal-chelating section, TG and TO were predicted to have really low antibacterial activity because they had weak chelating activity. Surprisingly, only TG and TO could inhibit *E. coli* at a concentration of 2.5 mg/mL (*p* < 0.05), and only TO could wipe out *Pseudomonas* at two concentrations of 2.5 and 1.25 mg/mL (*p* < 0.05), while for the others, the whole population came back right after they came in contact with nutrients. Hence, TO could have a different mechanism of action against these Gram-negative bacteria, such as membrane leakage. Iron is one of the most essential elements required by major microorganisms since it is a cofactor working along with many important enzymes and cytochromes to generate energy (e.g., ATP, NADPH) [41]. The chelation of metals does not just indicate the antioxidant power; it also contributes to the explanation for the inhibition of microorganisms. It was proposed that the lethal impact came from the number of hydroxyl groups that can shift between the portion of saturated and unsaturated lipids on the cell membrane, causing cell destruction [42]. *E. coli* alone has been reported to have seven different ways to acquire iron, even in iron deficiency conditions [43]. By withholding iron molecules, tannins can cause energy depletion and lead to cell death. This is the main mode of defence against microorganisms commonly found in plants. Plants accumulate tannins in the wood to help them fight many wood-decaying fungi such as *Gloeophyllum tmheum*, *Coriolus versicolor*, *Fomes annosus*, *Poria monticola*, *Merulius lacrymans*, and *Trametes hirsuta* [44].

### 3.3. Effect of TO and TVG on Vacuum-Packed Beef Mince during Refrigerated Storage

TO and TVG were selected for the treatment of beef mince owing to their higher antioxidant and antibacterial activities. 

#### 3.3.1. Chemical Analyses

pH

The pH values of all meat samples on the last day of refrigerated storage are shown in Table 3. Beef mince treated with sodium metabisulphite showed a slightly higher pH value than the control and tannin-treated samples. However, there was no significant difference noted among all samples (*p* > 0.05). The recorded results agree with Yang et al. [45] and Istrati et al. [46] in that the pH values among treatments did not change so much (*p* > 0.05) and did not go above 5.3. The pH values of ground beef treated with lyophilised water extract of *Satureja hortensis* started from approximately 5.7 and changed drastically on day 21 [47,48]. In general, at low storage temperatures, meat that is exposed to air will break down faster, producing ammonia and amines, hence increasing the pH over time [49]. However, this trend of pH is not displayed in vacuumed meat. This could be due to how the bacteria flora grows in different air conditions. In vacuum conditions, the only bacteria that could grow is lactic acid, hence the drop in pH [50].

TBARS

The TBARS value measures the secondary oxidation product. As a result, when an antioxidant is used to extend the shelf life of meat, the TBA value of meat is expected to be lower, as the degradation is slower. At the start of the experiment, beef mince treated with SMS and TO had slightly lowered TBARS values compared to other treatments (Table 4). This indicated that SMS and TO are effective immediately after mixing with minced beef (*p* < 0.05). During the 7 days of storage, a slight decrease in the TBARS value was reported in all meat samples. Whereas on day 14, again a slight increase, and on the last day of storage, a slight decrease, was noted in all meat samples. This increase and decrease in secondary oxidation products can be explained by the leaching out of the effect of the primary oxidation product during storage [51]. Nevertheless, TVG (0.25 or 0.5%)-treated samples showed a similar rate of lipid degradation to the control throughout the storage study (*p* > 0.05). In general, vacuum-packed beef steak showed lowered TBARS values compared to polyvinyl chloride overwrap packaging [24]. Smaoui and others [48] found that the combination of essential peppermint oil and BacTN635 had the same TBA value as the control on the first day. Moreover, the application of different plants such as oregano, thyme, lemon balm, marjoram, and summer savoury did not lower the TBA value right after the marination [46,47]. Nevertheless, if the preservatives are active enough, a strong impact on the TBA value will be made. For instance, sodium metabisulphite decreased the TBA value from 1.71 to 0.94 on the first day. Regarding the initial input, most of the tannin trials were able to maintain organic structures better than some commercial products, such as ActivinTM, Pycnogenol^®^ [52,53], ongoing research products, date pit extract [54], garlic straw [55], and lotus extracts [56]. This result indicates that treatment with TO and vacuum packaging showed a combined effect in lowering the oxidation and peroxidation of products during refrigerated storage.

Metmb

The formation of metmyoglobin in vacuum-packed minced beef treated with TO and TVG during refrigerated storage is presented in Table 4. Meat colour is defined by the myoglobin state on the meat surface. During the 21-day observation, it was true that the colour only changed on the surface. Even though the meat used in this research was minced, which means oxygen should be spread all over the meat body, only around 1 mm of the meat surface turned brown after being exposed to the air for so long. A simple explanation was that in the process of vacuum packaging, oxygen molecules were removed. When the bag is opened, oxygen molecules in the air interact with ferrous deoxymyoglobin, forming ferric metmyoglobin, which becomes brown with an undesirable colour [57]. There have not been any legally made documents about colour acceptability because general acceptance may vary depending on seasons [58] as well as flavour intensity [59]. It was suggested that customers’ rejection started from 30% and sharply dropped at 40% Metmb. [60]. Mohan et al. [61] suggested that their products received negative evaluations when containing over 40% Metmb. Observations from our experiment indicated an interesting behaviour trend of myoglobin. In the beginning, all meat samples had a very distant starting point, ranging from 46% to 79% (*p* < 0.05). Except for the control, all treatments increased by 8 to 40% by day 7. They all had a significant downfall on day 14 before they levelled off for the rest of the trial (*p* < 0.05). If the threshold of 30% is drawn, samples with 0.25% or 0.5% TVG, SMS, and even control are acceptable on the last day of the 21-day trial. Similar data were recorded for beef patties by Hayes et al. [62] and O’ Gardy, Monahan, and Brunton [63] that the percentage of Metmb was higher on the first day and lower on the last day. In addition, recently, Reyes et al. [24] found that beef steak packed under a vacuum showed decreased Metmb values, while steak packed under polyvinyl chloride overwrap showed an increase in Metmb values during 35 days of storage in a refrigerator. 

#### 3.3.2. Colour

The instrumental colour value of vacuum-packed beef mince treated with TO and TVG on day 21 is shown in Table 3. It was noted that all colour values including L* (lightness), a* (redness), b* (yellowness), and ΔE (total colour difference) varied among the samples during storage. In general, the sample treated with SMS showed less colour difference (lowered ΔE) throughout the storage compared to other treatments (*p* < 0.05). At the end of the storage, there was no significant difference noted in the L* and a* values of all samples (*p* > 0.05), whereas a high b* value was reported in the sample treated with a higher concentration of TO and TVG (0.5%) compared to other treatments. The increase in yellowness could be the adverse effect of a higher concentration of TO and TVG. However, when the total colour difference was calculated, it was found that only the SMS-treated sample had a lower value, which is closer to the standard colour value. Nevertheless, no significant difference in ΔE was reported between the control and sample treated with TO and TVG. These results agree with the Metmb values of SMS-treated samples. However, no relation was observed between the Metmb value and ΔE value of the control and samples treated with TO and TVG. Interestingly, the ΔE value of all samples at the end of the storage day was under the acceptable limit. Reyes et al. [24] reported that beef steak packed under a vacuum had higher L* and a* values compared to the meat packed under polyvinyl chloride.

#### 3.3.3. Microbial Analysis

Besides the chemical deterioration, the microflora in fresh meat is another important factor that decides whether meats are safe for consumption or not. Microbial changes in the food sample could accelerate chemical deterioration during refrigerated storage [32]. Generally, fresh meat can last around 7 days until it is eliminated by microbial spoilage [64]. The initial total viable count of minced beef in all trials started with a really high number in the range of 6.22–6.88 CFU/g. In a survey in the US in 2005, it was reported that the mean log10 of the biota of fresh ground beef was 6.7 [65]. It is safe to assume that a TVC value around 6.7 is normal. As the day of storage increased, the TVC value of all samples gradually increased, except for the sample treated with SMS. The lowest TVC value was noted in the sample treated with SMS at the end of the storage (Table 5). However, the continuous increase in the TVC value of the meat sample treated with TO and TVG is in contrast with the antibacterial activity of tannins and needs further investigation. On the other hand, when the H2S-producing bacterial count was determined, a gradual increase in the count was noted in the control sample compared to the other treatment (*p*<0.05). Nevertheless, treatment with SMS, TO, and TVG significantly lowered the H2S-producing bacterial count during storage (*p*<0.05). At the end of storage, the SMS-treated sample showed a 3.30 CFU/g value, whereas the TO- and TVG-treated samples (0.25 and 0.5%) showed less than 3 CFU/g. H2S-producing bacteria in any food sample are responsible for the foul smell indicating the spoilage of the food. Nevertheless, both the TVC and the H2S-producing bacteria count of the samples treated with SMS, TO, and TVG showed good microbiological quality based on the standards established in Australia and New Zealand [66].

## 4. Conclusions

Tannin samples extracted from different sources showed different total phenolic contents. The highest TPC values were observed in the order of TVG > TG > TE > TO (*p* < 0.05). However, the antioxidant activities of all four samples do not correlate with the TPC values of each sample. TO had the highest DPPH radical scavenging activity. Similarly, TO and TG showed the highest reducing power. Metal-chelating activity was highest for TE and lowest for TG. For antibacterial activity, the trend again fluctuated. All samples inhibited 100% of L. monocytogens growth at a minimum concentration of 1.25 mg/mL. Only TE and TVG could inhibit 100% Staphylococcus at 2.5 mg/mL. TO, TE, and TVG could inhibit more than 80% of Pseudomonas population at 1.25 mg/mL, but the bactericidal test confirmed that only TO could kill Pseudomonas from 1.25 mg/mL. TG and TO could prohibit E. coli from growing from 1.25 mg/mL, but only at 2.5 mg/mL did they exhibit the bactericidal effect. When vacuum-packed beef mince was treated with TO and TVG at two different concentrations, 0.25 and 0.5%, the chemical and microbial changes were retarded as compared to the control sample. Regarding lipid oxidation, TO was more effective in all treatments. In the aspects of colour and oxymyoglobin oxidation, TVG treatment was more effective than the other treatment. However, H2S-producing bacterial contamination was controlled better by TO and TVG than by the control and SMS. Therefore, TO and TVG could be used as promising natural food preservatives to control oxidation and microbial contamination.

## Figures and Tables

**Table 1:** Total phenolic contents and DPPH radical scavenging activity (IC50 value) of different tannin solutions.

Extract	TPC (g GAE/100 g Powder)	IC50 (µg/mL)
TO	35.82 ± 1.91 d	4.03 ± 0.08 d
TE	51.75 ± 1.19 c	20.25 ± 1.02 a
TVG	71.25 ± 4.04 a	15.26 ± 0.76 b
TG	61.14 ± 0.53 b	6.23 ± 0.06 c

Mean ± SD (*n* = 6). Means with different letters in columns are significantly different (*p* < 0.05).

**Table 2:** Percentage of inhibition of Gram-positive and Gram-negative bacteria by tannin samples at concentrations of 1.25 and 2.5 mg/mL.

Bacteria	TG (mg/mL)		TO (mg/mL)		TE (mg/mL)		TVG (mg/mL)	
	1.25	2.5	1.25	2.5	1.25	2.5	1.25	2.5
***L. monocytogenes***	100 ± 4.0 a	100 ± 9.1 a	100 ± 2.8 a	100 ± 5.6 a	100 ± 3.7 a	100 ± 0.9 a	100 ± 0.6 a	100 ± 9.2 a
***S. aureus***	0.0 ± 0.0 c	42.4 ± 3.6 b	0.0 ± 0.0 a	56.4 ± 2.0 b	68.6 ± 0.8 c	100 ± 6.2 a	100 ± 3.4 a	100 ± 4.1 a
***E. coli***	99 ± 1.8 a	100 ± 2.3 a	100 ± 1.3 a	100 ± 2.2 a	0.0 ± 0.0 d	34.9 ± 2.2 c	43.4 ± 8.2 b	54.4 ± 2.3 b
***P. aeruginosa***	36.3 ± 9.6 b	44.5 ± 5.3 b	100 ± 3.7 a	100 ± 4.1 a	82.7 ± 7.6 b	81.8 ± 2.3 b	97.4 ± 4.6 a	96.3 ± 5.6 a

Mean ± SD (*n* = 6). Means with different letters in columns are significantly different (*p* < 0.05).

**Table 3:** pH and colour properties of vacuum-packed beef mince treated with TO and TVG at the end of refrigerated storage.

	Control	SMS (0.05%)	TO (0.25%)	TO (0.5%)	TVG (0.25%)	TVG (0.5%)
pH	5.37 ± 0.03 a	5.65 ± 0.02 a	5.26 ± 0.01 ab	5.16 ± 0.00 b	5.31 ± 0.01 a	5.27 ± 0.01 ab
L*	47.69 ± 1.5 a	44.87 ± 0.6 a	43.24 ± 1.9 ab	43.05 ± 0.6 b	47.48 ± 0.4 a	46.45 ± 1.8 a
a*	15.09 ± 0.7 a	15.63 ± 0.8 a	15.16 ± 1.4 a	14.28 ± 0.5 a	14.89 ± 0.4 a	15.10 ± 0.8 a
b*	6.29 ± 0.4 c	5.92 ± 0.3 c	7.92 ± 0.2 b	9.17 ± 0.4 a	7.96 ± 0.3 b	9.01 ± 0.4 a
ΔE*	4.82 ± 1.6 a	2.24 ± 1.1 b	3.40 ± 1.0 ab	3.58 ± 0.6 ab	3.14 ± 1.5 ab	3.60 ± 1.5 ab

Mean ± SD (*n* = 6). Means with different letters in a row are significantly different (*p* < 0.05). L—brightness; a—redness; b—yellowness; * the difference between day 1 and day 21; ∆E = $\sqrt{{\left(L_{2}-{\;L}_{1}\right)}^{2}+{\left(a_{2}-a_{1}\right)}^{2}+{\left({\;b}_{2}-{\;b}_{1}\right)}^{2}}$, where L_1_, a_1_, and b_1_ denote values at 0 days of storage time, and L_2_, a_2_, and b_2_ denote values at 21 days of storage time.

**Table 4:** TBARS and Metmb (%) values of vacuum-packed beef mince treated with TO and TVG during refrigerated storage.

Samples	TBARS (mg Malonaldehyde/kg Sample)				Metmb (%)			
	0-Day	7-Day	14-Day	21-Day	0-Day	7-Day	14-Day	21-Day
**Control**	1.71 ± 0.1 a	0.98 ± 0.1 ab	1.14 ± 0.3 a	1.46 ± 0.1 a	69.49 ± 3.5 ab	44.54 ± 3.5 d	1.58 ± 0.8 c	2.97 ± 1.9 d
**SMS (0.05%)**	1.04 ± 0.3 ab	0.89 ± 0.1 ab	0.68 ± 0.0 b	0.85 ± 0.1 ab	46.83 ± 1.4 c	56.49 ± 2.1 c	2.93 ± 0.9 c	16.56 ± 0.6 c
**TO (0.25%)**	1.02 ± 0.1 b	0.49 ± 0.0 b	0.98 ± 0.2 ab	0.81 ± 0.0 ab	78.08 ± 3.8 a	118.38 ± 6.4 a	65.00 ± 3.2 a	66.19 ± 3.6 a
**TO (0.5%)**	1.51 ± 0.1 a	0.46 ± 0.1 b	0.75 ± 0.0 b	0.53 ± 0.0 b	79.25 ± 3.9 a	114.14 ± 2.9 a	66.44 ± 1.5 a	67.33 ± 3.5 a
**TVG (0.25%)**	1.51 ± 0.1 a	1.28 ± 0.0 a	1.50 ± 0.1 a	1.08 ± 0.0 a	63.67 ± 4.1 b	81.86 ± 0.5 b	24.77 ± 0.5 b	23.56 ± 2.3 b
**TVG (0.5%)**	1.8 ± 0.0 a	1.61 ± 0.3 a	1.85 ± 0.2 a	1.42 ± 0.3 a	73.71 ± 5.6 a	81.22 ± 3.2 b	68.12 ± 2.2 a	24.06 ± 0.1 b

Mean ± SD (*n* = 6). Means with different letters in columns are significantly different (*p* < 0.05).

**Table 5:** TVC and H2S-producing bacteria count of vacuum-packed beef mince treated with TO and TVG during refrigerated storage.

Samples	TVC (CFU/g)				H2S-Producing Bacteria Count (CFU/g)			
	0-Day	7-Day	14-Day	21-Day	0-Day	7-Day	14-Day	21-Day
**Control**	6.22 ± 0.71 a	6.66 ± 0.15 a	6.86 ± 0.36 a	7.31 ± 0.08 a	3.96 ± 0.11 a	4.46 ± 0.11 a	4.58 ± 0.24 a	4.69 ± 0.55 a
**SMS (0.05%)**	6.57 ± 0.01 ab	6.57 ± 0.20 a	6.69 ± 0.13 a	6.39 ± 0.12 b	3.74 ± 0.19 a	3.80 ± 0.14 b	3.30 ± 0.00 b	3.30 ± 0.00 b
**TO (0.25%)**	6.67 ± 0.12 a	6.93 ± 0.09 a	7.42 ± 0.11 a	7.56 ± 0.20 a	4.26 ± 0.08 a	3.99 ± 0.12 b	3.60 ± 0.00 b	2.47 ± 0.19 c
**TO (0.5%)**	6.76 ± 0.06 a	6.81 ± 0.03 a	7.30 ± 0.14 a	7.40 ± 0.14 a	4.11 ± 0.05 a	4.00 ± 0.00 ab	3.24 ± 0.34 b	2.11 ± 0.22 c
**TVG (0.25%)**	6.88 ± 0.13 a	7.22 ± 0.60 a	7.06 ± 0.03 a	7.51 ± 0.09 a	3.93 ± 0.08 a	4.14 ±0.12 a	4.03 ±0.23 a	2.39 ± 0.05 c
**TVG (0.5%)**	6.66 ± 0.13 a	6.76 ± 0.07 a	7.33 ± 0.01 a	7.50 ± 0.04 a	3.93 ± 0.39 a	4.20 ± 0.11	4.10 ± 0.14 a	2.21 ± 0.11 c

Mean ± SD (*n* = 6). Means with different letters in columns are significantly different (*p* < 0.05).

## Data Availability

The data presented in this study are available on request from the corresponding author.
